# Associations between plasma protein, IgG and IgA N-glycosylation and metabolic health markers in pregnancy and gestational diabetes

**DOI:** 10.1371/journal.pone.0284838

**Published:** 2023-04-20

**Authors:** Tamara Štambuk, Domagoj Kifer, Lea Smirčić-Duvnjak, Marijana Vučić Lovrenčić, Olga Gornik

**Affiliations:** 1 Department of Biochemistry and Molecular Biology, Faculty of Pharmacy and Biochemistry, University of Zagreb, Zagreb, Croatia; 2 Genos Glycoscience Research Laboratory, Zagreb, Croatia; 3 Vuk Vrhovac University Clinic for Diabetes, Endocrinology and Metabolic Diseases, Merkur University Hospital, University of Zagreb School of Medicine, Zagreb, Croatia; Gachon University Gil Medical Center, DEMOCRATIC PEOPLE’S REPUBLIC OF KOREA

## Abstract

**Background:**

Monitoring human circulating N-glycome could provide valuable insight into an individual’s metabolic status. Therefore, we examined if aberrant carbohydrate metabolism in gestational diabetes mellitus (GDM) associates with alterations in plasma protein, immunoglobulin G (IgG) and immunoglobulin A (IgA) N-glycosylation.

**Methods:**

Plasma protein, IgG and IgA N-glycans were enzymatically released, purified and chromatographically profiled in 48 pregnant women with normal glucose tolerance and 41 pregnant women with GDM, all sampled at 24–28 weeks of gestation. Linear mixed models adjusting for age and multiple testing (FDR<0.05) were used to investigate the associations between glycosylation features, metabolic markers and GDM status.

**Results:**

Fasting insulin exhibited significant associations to numerous glycan traits, including plasma protein galactosylation, sialylation, branching, core fucosylation and bisection, to IgG core fucosylated, bisected (FA2B) and afucosylated disialylated (A2G2S2) glycan and to IgA trisialylated triantennary (A3G3S3) glycan (p_adj_ range: 4.37x10^-05^–4.94x10^-02^). Insulin resistance markers HOMA2-IR and HOMA2-%B were mostly associated to the same glycan structures as fasting insulin. Both markers showed positive association with high-branched plasma glycans (p_adj_ = 1.12x10^-02^ and 2.03x10^-03^) and negative association with low-branched plasma glycans (p_adj_ = 1.21x10^-02^ and 2.05x10^-03^). Additionally, HOMA2-%B index was significantly correlated with glycosylation features describing IgG sialylation. Multiple plasma protein IgG and IgA glycans showed significant associations with total cholesterol and triglyceride levels. None of the tested glycan traits showed a significant difference between GDM and normoglycemic pregnancies.

**Conclusion:**

Markers of glucose homeostasis and lipid metabolism in pregnancy show extensive associations to various N-glycosylation features. However, plasma protein, IgG and IgA N-glycans were not able to differentiate pregnant women with and without GDM, possibly due to numerous physiological changes accompanying pregnancy, which confound the impact of GDM on protein glycosylation.

## Introduction

Gestational diabetes mellitus (GDM) is defined as a diabetes diagnosed in the second or third trimester of pregnancy that was not clearly overt diabetes prior to gestation [1]. Globally, there is a lack of universally accepted diagnostic protocols or set of diagnostic criteria for GDM, however, diagnosis is usually performed using an oral glucose tolerance test (OGTT), with glucose threshold levels defined according to IADPSG or WHO 2013 criteria [2, 3]. Due to the lack of overall consensus on diagnostic criteria it is also hard to estimate GDM prevalence, but available reports suggested that the condition affects from 1% to >30% of pregnancies [4].

The metabolic abnormalities characterizing GDM include increased insulin resistance and β-cell defects, that likely exist even before conception in many cases [5, 6]. Essentially, human pregnancy represents a considerable metabolic stress, characterized by a series of extensive metabolic changes. For instance, basal endogenous glucose production increases by 30%, whereas fasting glucose concentrations decrease due to increased glucose utilization by fetus. Additionally, peripheral insulin sensitivity decreases up to 50% and insulin secretion increases 2-3-fold to compensate for decreased insulin sensitivity [6]. In women that develop GDM the insulin response becomes inadequate with the advancing pregnancy, as the insulin resistance increases, which leads to hyperglyceamia. GDM is currently the most common medical complication of pregnancy, increasing the risk of both short-term (pregnancy-related hypertensive disorders, excess fetal growth and adiposity) and long-term complications (obesity, impaired glucose metabolism and premature cardiovascular disease) in both mother and infant [6, 7]. In fact, it was reported that women with GDM have a 7-fold higher risk of type 2 diabetes than women with normoglycemic pregnancies, making GDM the best-known risk factor for type 2 diabetes development [8]. Attempts to prevent GDM through lifestyle, dietary or medical interventions have not shown consistent benefits, and none could be recommended for routine use, making an optimal long-term management of mother and infant challenging.

Protein N-glycosylation is highly regulated, enzymatic, complex and multistep process involving numerous glycosyltransferases and glycosidases that determine the position and structure of assembled N-glycans [9]. Glycosylation is known to reflect the physiological state of an organism and changes thereof [10], due to responsiveness of glycosylation machinery to numerous cellular and environmental stimuli. Since glycosylation has been implicated in various physiological and pathological processes [11], glycans represent potentially valuable reporters of current health and future disease risk.

Accumulating evidence is demonstrating that protein N-glycosylation participates in various processes involved in the regulation and maintenance of glucose homeostasis [12] and it is tightly connected to the nutrient sensing through the hexosamine biosynthetic pathway [13]. Moreover, N-glycosylation alterations have been observed in all major diabetes subtypes, including GDM. However, due to its complexity and discrepancies shadowing its diagnosis, number of studies investigating changes of N-glycosylation in this condition is limited. Most of glycomics research related to GDM has been focusing on various secretory glycoproteins, including human chorionic gonadotropin [14], glycodelin-A [15] and immunomodulatory proteins from human milk—secretory immunoglobulin A (sIgA) and lactoferrin [16].

Monitoring of human circulating N-glycome can provide valuable insights into an individual’s metabolic status. Thus, in search of potential biochemical markers of GDM, we aimed to examine if aberrant carbohydrate metabolism associates with alterations in plasma protein, immunoglobulin G (IgG) and immunoglobulin A (IgA) N-glycosylation.

## Methods

### Study population

The study included residual samples from 89 pregnant women, all routinely referred to our tertiary health-care facility between 24 and 28 weeks of gestation (median 25 weeks), to screen for undiagnosed hyperglycaemia in pregnancy, according to the WHO-2013 recommended procedure for the classification of hyperglycaemia first detected in pregnancy. Diagnostic workup was carried out in the morning, after an overnight fast, by peroral ingestion of glucose (75 g) and venous blood sampling immediately before, and 1 and 2 hours after glucose load.

### Routine laboratory tests

Glucose was measured with an enzymatic hexokinase method (BC-AU680, Beckman Coulter, USA) in fasting, 1-h and 2-h venous plasma samples within 1 hour from the sampling, to avoid possible interference of in vitro glycolysis. The same biochemical platform was used for the measurement of routine laboratory tests: total and HDL-cholesterol, triglycerides, urate, AST, ALT, GGT and total proteins in fasting samples. HbA1c was determined with an NGSP-certified/IFCC traceable immunoturbidimetric assay (Tina-quant HbA1c Gen 3; Cobas Integra, Roche Diagnostics, Switzerland), and fructosamine with NBT colorimetric test on the same analytical platform. Fasting insulin was measured by an automated chemiluminescence immunoassay traceable to WHO 1st IRP 66/304 standard (Advia Centaur XP, Siemens Healthineers, USA). Homeostasis Model Assessment HOMA2 Calculator (version 2.2.2, Diabetes Trials Unit, University of Oxford, available at http://www.dtu.ox.ac.uk/homacalculator/index.php, was used to estimate beta cell function [HOMA2-B (%)] and insulin sensitivity [HOMA2-IS (%)] from fasting glucose and insulin concentrations [17].

This study was approved by the Ethics Committee of the Merkur University Hospital (Approval No. 0311-2172/3, 21.2.2019) as a part of the Diagnostic evaluation of potential biochemical markers of diabetes study, whereby residual biological material and routinely collected medical data from regular medical check-up is used. Basic characteristics of the study population are given in Table 1.

**Table 1:** Characteristics of study population^a^.

Variable	Control	Case	p-value^b^
N	48	41	
age (years)	32 (30; 35)	31 (28; 37)	0.665
BMI (kg/m2)	25.0 (22.8; 26.6)	26.6 (23.5; 31.6)	< 0.001
fasting glucose (mmol/L)	4.7 (4.5; 4.9)	5.1 (4.9; 5.2)	< 0.001
HbA1c (mmol/mol)	29 (27; 32)	31 (29; 33)	0.07
HbA1c (%)	4.8 (4.7; 5.1)	5.0 (4.8; 5.2)	0.095
fasting insulin (pmol/L)	43.5 (34.0; 61.3)	60.9 (44.3; 78.5)	0.012
HOMA2-IR	0.925 (0.721; 1.293)	1.282 (1.000; 1.689)	0.007
HOMA2-%B	107.7 (88.5; 132.4)	115.5 (92.5; 136.1)	0.487
triglycerides (mmol/L)	1.66 (1.22; 2.06)	2.12 (1.60; 2.61)	0.004
HDL (mmol/L)	1.785 (1.500; 2.055)	1.620 (1.500; 1.850)	0.101
total cholesterol (mmol/L)	6.52 (5.59; 7.73)	6.45 (5.54; 7.22)	0.639
total proteins (g/L)	64 (62; 67)	65 (63; 67)	0.365
urate (μmol/L)	201 (180; 240)	199 (168; 259)	0.869
ALT (U/L)	11.5 (8.0; 16.0)	10.0 (7.0; 12.0)	0.135
AST (U/L)	22.5 (17.0; 26.3)	18.0 (16.0; 22.0)	0.002
GGT (U/L)	9.5 (8.0; 13.0)	8.0 (7.0; 10.0)	0.098
fructosamine (μmol/L)	184.0 (179.8; 195.5)	187.0 (175.0; 198.0)	0.905

^a^values are given as median (interquartile range)

^b^p-values were calculated using Wilcoxon rank sum test (significance level α = 0.05)

### N-glycan analysis

#### IgG capturing from human plasma

Human polyclonal IgG was captured from 70 μL of human plasma by affinity chromatography using protein G monolithic plate (Bia Separations, Slovenia), as described previously [18]. In brief, plasma samples were diluted 7x with phosphate-buffered saline (PBS, Merck, Germany) and transferred to 96-well protein G plate, then immediately washed with PBS. In the final step, IgG was eluted with 1 mL of 0.1 M formic acid (Merck, Germany) and instantly neutralized with 170 μL of 1 M ammonium bicarbonate (Across Organics, USA). Finally, an aliquot of 300 μL of IgG eluate was dried by vacuum centrifugation and used in further analytical processes.

#### IgA capturing from human plasma

Human polyclonal IgA was captured from 40 μL of human plasma by affinity chromatography using CaptureSelect IgA affinity matrix (Thermo Fischer Scientific, MA, USA). Twenty-five μL of IgA affinity matrix bead slurry was applied to the each well of a 96-well Orochem filter plate (Orochem Technologies Inc., Il, USA). The beads were prewashed with water and 4x with PBS on a vacuum manifold. Plasma samples were diluted 7x with PBS, transferred to Orochem plate containing washed beads and incubated 30 min on a plate shaker. The beads with captured IgA were then washed 3x with PBS. In the final step, IgA was eluted with 200 μL of 0.1 M formic acid and instantly neutralized with 34 μL of 1 M ammonium bicarbonate. An aliquot of 100 μL of IgA eluate was dried by vacuum centrifugation and used in further analytical processes.

#### Enzymatic release of plasma protein, IgG and IgA N-glycans

Denaturation of plasma samples (10 μl) was performed using 20 μl of 2% (w/v) SDS (Invitrogen, USA) followed by incubation at 65°C for 10 min. Dried IgG and IgA samples were resuspended in 30 μl of 1.33% (w/v) SDS followed by incubation at 65°C for 10 min. Next, 10 μl of 4% (v/v) Igepal-CA630 (Sigma Aldrich, USA) was added to all denatured samples. N-glycans were enzymatically cleaved from glycoproteins by adding 1.2 U of PNGase F (Promega, USA) to the mixture followed by overnight incubation at 37°C.

#### Fluorescent labelling and HILIC-SPE purification of plasma protein, IgG and IgA N-glycans

Released plasma protein and IgG N-glycans were labelled with a fluorescent dye 2-aminobenzamide (2-AB). The labelling mixture consisted of 2-AB (19.2 mg/ml; Sigma Aldrich, USA) and 2-picoline borane (44.8 mg/ml; Sigma Aldrich, USA) in dimethyl sulfoxide (Sigma Aldrich, USA) and glacial acetic acid (Merck, Germany) mixture (70:30 v/v). Released IgA N-glycans were labelled with a fluorescent dye procainamide (ProA). The labelling mixture consisted of ProA (38.3 mg/ml; Sigma Aldrich, USA) and 2-picoline borane (44.8 mg/ml) in dimethyl sulfoxide and glacial acetic acid mixture (70:30 v/v). The procedure was identical for all samples henceforward; total volume of 25 μl of the labelling mixture was added to each sample, followed by a 2h incubation at 65°C. Purification of labelled glycans was performed by hydrophilic interaction liquid chromatography solid-phase extraction (HILIC-SPE) on a 0.2 μm GHP filter plate (Pall Corporation, USA). Following the incubation, the samples were brought to 96% acetonitrile (ACN) by adding 700 μl of ACN (J.T. Baker, USA) and applied to each well of the GHP filter plate. Solvent was removed by application of vacuum using a vacuum manifold. All wells were prewashed with 70% ethanol (Sigma-Aldrich, USA) and water, followed by equilibration with 96% ACN. Loaded samples were subsequently washed 5× with 96% ACN. N-glycans were eluted with water and stored at -20°C until further analysis.

#### Chromatographic profiling and structure assignment of plasma protein, IgG and IgA N-glycans

**Fig 1:**
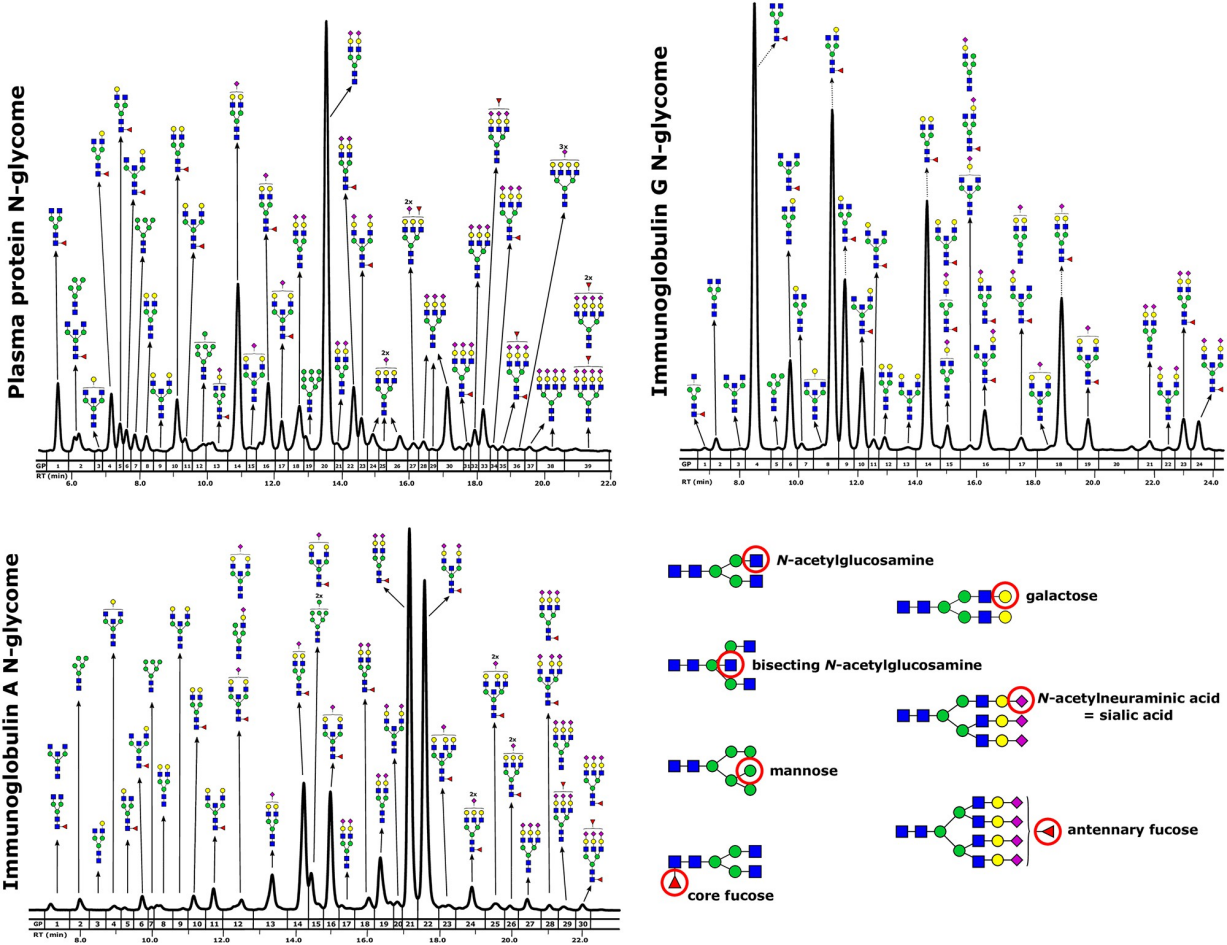
Representative HILIC-UPLC-FLR chromatograms of plasma protein, immunoglobulin G and immunoglobulin A N-glycomes, with graphic representation of the most abundant glycan structure corresponding to each glycan peak (GP).

Fluorescently labelled N-glycans were separated by hydrophilic interaction liquid chromatography (HILIC) on Acquity ultra performance liquid chromatography (UPLC) H-Class instrument (Waters, USA). The instrument consisted of a quaternary solvent manager (QSM), a sample manager (SM) and a fluorescence (FLR) detector, and was controlled by Empower 3 software build 3471 (Waters, USA). Excitation wavelength was set at 250 nm or 310 nm and emission wavelength at 428 nm or 370 nm for 2-AB and ProA labelled glycans, respectively. For N-glycan separation BEH Glycan chromatography column was used (Waters, USA). System was calibrated with an external standard containing hydrolysed and labelled glucose oligomers, which were used for the conversion of individual glycans’ retention times to glucose units (GU). Glycan structures were assigned using MS/MS approach, using HILIC-UPLC coupled to Compact ESI-QTOF-MS system via Ion Booster ion source (Bruker Daltonics, Germany). The MS instrument was controlled by Hystar software version 3.2 (Bruker Daltonics), and operated in a positive ion mode, with capillary voltage set to 2250 V and nebulizing gas pressure of 5.5 Bar. Drying gas (nitrogen) was applied to source at a flow rate of 4 L/min and temperature of 150°C, while vaporizer temperature was set to 200°C and flow rate of 5 L/min. Nitrogen was used as a source gas, while argon was used as a collision gas. Spectra were recorded in m/z range of 150–4000 at 0.5 Hz frequency. MS/MS analysis was performed using Auto MS/MS mode, which selects three precursors with the highest intensities for CID fragmentation. Glycan compositions and structural features were assigned using DataAnalysis, GlycoWorkbench and Glycomode software tools, based on obtained MS and MS/MS spectra. For quantitative analysis, the fluorescence chromatograms were all separated in the same manner into 39 glycan peaks (GP1–GP39) for plasma protein N-glycome, 24 peaks (GP1-GP24) for IgG N-glycome and 30 peaks (GP1-GP30) for IgA N-glycome (Fig 1). List of N-glycan structures corresponding to individual glycan peak is available in S1 Table. The amount of glycans in each peak was expressed as a percentage of the total integrated area. Specific glycosylation features, including sialylation, fucosylation, bisection, galactosylation and degree of glycan branching were described using derived traits, which are calculated from initial glycan peaks. Sixteen derived traits were calculated to describe plasma protein N-glycome, 9 to describe IgG N-glycome and 12 to describe IgA N-glycome (S2 Table).

#### Statistical analysis

Medians with interquartile ranges (IQR) were calculated for variables presented in Table 1. Non-parametric statistics (Wilcoxon rank sum test with continuity correction) was used for comparison between the groups (presence or absence of GDM), with significance level α = 0.05.

Area under each glycan peak was normalized by total chromatogram area and expressed as a percentage. Obtained percentages were logit transformed and used to calculate derived glycan traits (S2 Table).

General linear models were used to estimate the associations between the glycan traits and metabolic markers. Glycan trait was modelled as a dependent variable, metabolic marker as independent variable, while participant’s age was used as a covariate. Before model construction, both glycan and metabolic marker values were transformed by inverse transformation of ranks to the standard normal distribution.

Differences in particular glycan trait between subjects with and without gestational diabetes were estimated by general linear modelling as well. Glycan trait was modelled as a dependent variable, and GDM status (control or case) as an independent variable. Age was again modelled as a covariate. Before model construction, glycan data were transformed by inverse transformation of ranks to the standard normal distribution. All calculated p-values were gathered and adjusted using Benjamini-Hochberg method modified by Li and Ji to control false discovery rate at level alpha = 0.05. Effective number of independent tests reduced to the 205 tests (from 1032 total tests) based on the correlation between tested glycan traits.

Statistical analysis was performed in R programming software for statistical computing [19].

## Results

### Association between plasma protein N-glycosylation and metabolic markers in pregnancy

**Fig 2:**
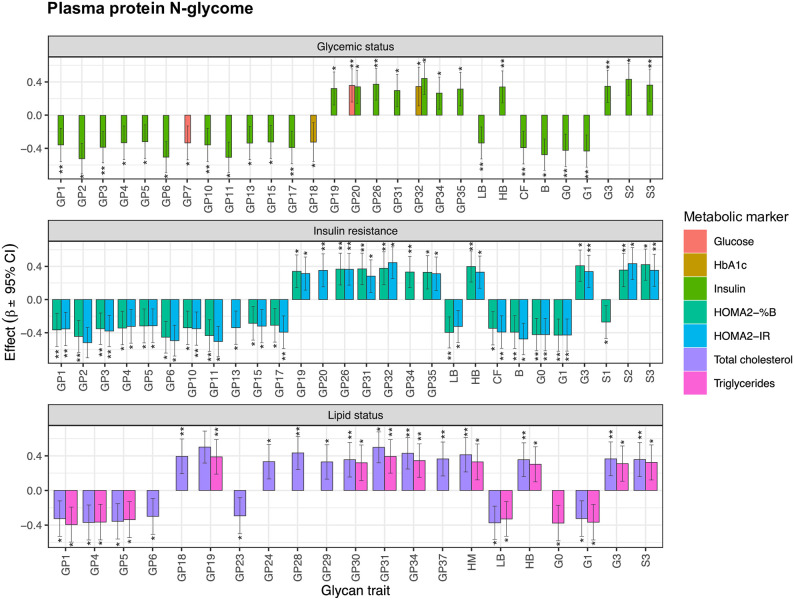
Significant associations of plasma protein N-glycan traits with metabolic parameters in pregnancy. Effect size represents beta coefficient estimated through regression model. Error bars represent 95% confidence intervals. B—bisecting GlcNAc; CF—core fucosylation; GP—glycan peak; G0 –agalactosylation; G1 –monogalactosylation; G3 –trigalactosylation; HB—high branching; HM—high mannose; LB—low branching; S0 –asialylation; S1 –monosialylation; S2 –disialylation; S3 –trisialylation;; *–adjusted p-value in range 0.05–0.01; **–adjusted p-value in range 0.01–0.001; ***–adjusted p-value < 0.001.

To examine the link between plasma protein N-glycosylation and metabolic markers in pregnancy a regression analysis was performed, using a general linear model. Numerous and extensive associations were found between plasma protein N-glycan traits and metabolic markers, that are visualized in Fig 2 and listed in S3 Table.

From the markers estimating an individual’s glycemic status, fasting insulin has demonstrated the most significant associations to various glycan traits, such as GP2 (structures FA2B and M5, p_adj_ = 4.37x10^-05^), GP11 (structure FA2BG2, p_adj_ = 4.68x10^-05^), GP6 (structure FA2 [6] BG1, p_adj_ = 6.19x10^-05^) and incidence of bisecting GlcNAc (B, p_adj_ = 2.27x10^-04^), among many other. Fasting glucose was significantly associated with GP20 (structure A2G2S2, p_adj_ = 8.87x10^-03^) and GP7 (structures M6 and FA2 [3] BG1, p_adj_ = 1.50x10^-02^), while HbA1c was significantly related to GP32 (A3G3S3, p_adj_ = 3.12x10^-02^) and GP18 (A2G2S2, p_adj_ = 4.94x10^-02^).

Due to numerous significant associations between glycans and insulin levels, we also aimed to investigate the potential relationship between insulin resistance and N-glycosylation. Again, glycan traits the most prominently related to HOMA2-IR index were GP2, GP11, GP6 and incidence of bisecting GlcNAc (range p_adj_ = 4.37x10^-05^–2.27x10^-04^). Additionally, HOMA2-%B index was also significantly associated to these same glycan structures (range p_adj_ = 6.29x10^-04^–3.85x10^-03^). Both HOMA2-IR and HOMA2-%B have also shown significant positive association with highly branched glycan structures (HB, p_adj_ = 1.12x10^-02^ and 2.03x10^-03^, respectively) and negative association with low branched glycan structures (LB, p_adj_ = 1.21x10^-02^ and 2.05x10^-03^, respectively), among many other (S3 Table).

The most distinctive associations between the markers describing an individual’s lipid status and glycan traits were the following: total cholesterol was significantly associated with glycans GP31 (FA3G3S3 and A3G3S3, p_adj_ = 4.68 x10^-05^), GP19 (M9, p_adj_ = 6.19x10^-05^), GP34 (FA3G3S3 and A4G4S3, p_adj_ = 6.42 x10^-04^) and high mannose structures (HM, p_adj_ = 2.49 x10^-03^). Triglyceride levels were most pronouncedly associated with glycans GP31 (FA3G3S3 and A3G3S3, p_adj_ = 3.29x10^-03^), GP1 (FA2, p_adj_ = 4.43x10^-03^), GP19 (M9, p_adj_ = 4.48x10^-03^) and agalactosylated glycans (G0, p_adj_ = 7.00x10^-03^).

### Association between IgG N-glycosylation and metabolic markers in pregnancy

**Fig 3:**
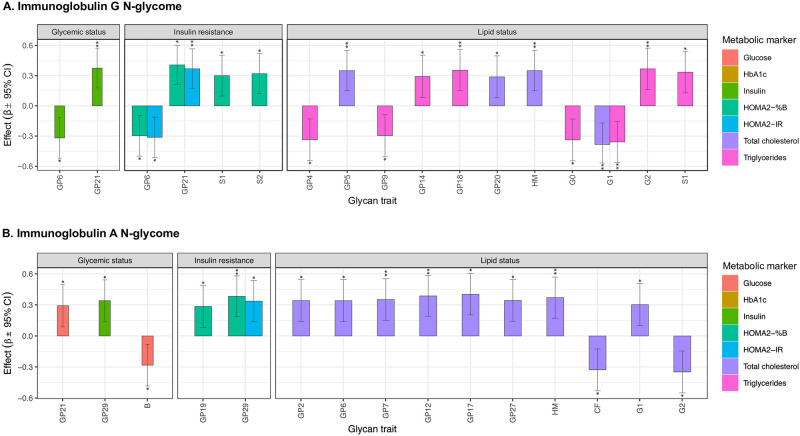
Significant associations of IgG and IgA N-glycan traits with metabolic parameters in pregnancy. Effect size represents beta coefficient estimated through regression model. Error bars represent 95% confidence intervals. B—bisecting GlcNAc; CF—core fucosylation; GP—glycan peak; G0 –agalactosylation; G1 –monogalactosylation; G2 –digalactosylation; HM—high mannose; S1 –monosialylation; S2 –disialylation; *–adjusted p-value in range 0.05–0.01; **–adjusted p-value in range 0.01–0.001; ***–adjusted p-value < 0.001.

Next, regression analysis was again performed to detect associations between circulating IgG N-glycosylation features and various metabolic markers in pregnancy. Multiple significant associations were detected between IgG N-glycan traits and metabolic markers, that are visualized in Fig 3 and listed in S4 Table.

From the markers estimating an individual’s glycemic status, only fasting insulin has demonstrated significant associations with GP21 (structure A2G2S2, p_adj_ = 5.32x10^-03^) and GP6 (structures FA2B and A2 [6] G1, p_adj_ = 1.83x10^-02^). Fasting glucose and HbA1c showed no significant associations with IgG glycosylation traits. Of the markers describing insulin resistance, HOMA2-IR index was found to be related to the same glycan structures as fasting insulin—GP21 and GP6 (p_adj_ = 6.572x10^-03^ and 2.08x10^-02^, respectively). HOMA2-%B index was also significantly associated to the same glycan structures (p_adj_ = 2.450x10^-03^ and 3.31x10^-02^, respectively), but it was also significantly related to the glycosylation features describing IgG sialylation (monosialylation, S1, p_adj_ = 3.03x10^-02^ and disialylation, S2, p_adj_ = 1.77x10^-02^).

Lastly, we examined the associations between IgG glycans and markers describing an individual’s lipid status. Total cholesterol was, among others, significantly associated with monogalactosylated (G1, p_adj_ = 4.82x10^-03^) and high mannose glycans (HM, p_adj_ = 9.24x10^-03^). Levels of triglycerides were most significantly associated with glycan traits describing IgG galactosylation—digalactosylation (G2, p_adj_ = 8.84x10^-03^), monogalactosylation (G1, p_adj_ = 9.24x10^-03^) and agalactosylation (G0, p_adj_ = 1.56x10^-02^). Complete list of all tested associations is provided in S4 Table.

### Association between IgA N-glycosylation and metabolic markers in pregnancy

Regression analysis was performed to identify associations between circulating IgA N-glycosylation features and various metabolic markers in pregnancy as well. Multiple significant associations were detected between IgA N-glycan traits and various metabolic markers, that are visualized in Fig 3 and listed in S5 Table.

IgA glycan traits showed several significant associations with markers of glycemic status and insulin resistance. Specifically, fasting glucose levels were linked to IgA glycan peak GP21 (structure FA2G2S2, p_adj_ = 4.05x10^-02^) and abundance of bisected glycans (B, p_adj_ = 4.47x10^-02^), whereas fasting insulin levels showed significant associations with GP29 (structure A3G3S3, p_adj_ = 1.12x10^-02^). HbA1c showed no significant associations with IgA glycans. Both HOMA2-IR and HOMA2-%B were found to be related to GP29 (structure A3G3S3, p_adj_ = 1.23x10^-02^ and p_adj_ = 4.48x10^-03^, respectively). HOMA2-%B also showed significant association with GP19 (structure A2G2S2, p_adj_ = 4.56x10^-02^).

Multiple IgA glycan traits showed significant associations with total cholesterol levels. Namely, the most prominently total cholesterol-associated IgA glycans were GP17 (structure A2G2S2, p_adj_ = 3.29x10^-03^), GP12 (structure A2BG1S1, p_adj_ = 4.36x10^-03^), high mannose glycans (HM, p_adj_ = 6.57x10^-03^) and digalactosylated glycans (G2, p_adj_ = 1.07x10^-02^). None of the tested IgA glycan traits were significantly correlated to triglyceride levels. Complete list of all investigated associations is provided in S5 Table.

### Association between plasma protein, IgG and IgA N-glycosylation and gestational diabetes

Since various significant associations between plasma protein, IgG and IgA N-glycosylation and metabolic health markers were observed, we examined differences in plasma protein, IgG and IgA N-glycan abundances between pregnancies with GDM and pregnancies with normal glucose tolerance using a general linear model. However, none of the initially measured glycan traits (glycan peaks) showed a significant difference between the groups (S6 Table and S1–S3 Figs in S1 File all adjusted p-values for plasma protein glycans > 0.23, IgG glycans > 0.40 and IgA glycans > 0.14).

We have also examined the associations of plasma protein, IgG and IgA derived glycan traits with gestational diabetes, by comparing GDM subjects to age-matched controls. Here as well, none of the examined derived glycan traits showed a significant difference between the groups (S6 Table and S1–S3 Figs in S1 File, all adjusted p-values for plasma protein glycans > 0.44, IgG glycans > 0.39 and IgA glycans > 0.51). Therefore, N-glycans cannot differentiate normoglycemic pregnancy from pregnancy affected by gestational diabetes.

### Impact of GDM on biochemical markers of intermediary metabolism

Fasting plasma glucose, triglycerides, as well as insulin and HOMA2-estimated insulin resistance were significantly higher in GDM, when compared to pregnant subjects with normoglycaemia (Table 1). Participants with GDM also had significantly higher BMI. Other biochemical markers, including HOMA2-estimated beta-cell function, were unchanged, while AST was lower in subjects with GDM.

## Discussion

GDM could be viewed as an imbalance between the required and achieved increase in insulin secretion during pregnancy [20].

In healthy women a prominent decrease in insulin sensitivity in the second and third trimester of pregnancy, responsible for impairment of insulin-dependent glucose uptake in peripheral tissues, represents an act of physiological adaption aiming to preserve carbohydrate supply for the fetus. It is attributed to increase in progesterone, estrogen, cortisol and human placental growth hormone and usually compensated by two to threefold increase in insulin secretion.

The twofold to threefold increase in insulin secretion seems to appear in early pregnancy and result from both an increase in number of islets and workload of beta cells. In addition, the increment in the glucose-induced first and second-phase insulin release was documented in the first trimester of pregnancy. GDM is characterized by an inadequate glucose dependent insulin secretion leading to hyperglycemia. As the most significant insulin resistance develops in the third trimester, factors leading to increase in insulin synthesis and secretion during early pregnancy might be independent of the decline of insulin sensitivity. Moreover, insulin resistance was shown not to be an important predictive factor for impaired glucose tolerance within 6 months after GDM. These results suggest the lack of increase in insulin secretion due to an impairment in beta cell function in early pregnancy as a prominent feature of GDM. In light of these hypothesis all potential contributors to beta cell dysfunction in early pregnancy are of significant scientific interest [21].

The impact of gestational diabetes on protein N-glycosylation is largely unknown. To the best of our knowledge, the current study is the first to examine plasma protein, IgG and circulating IgA N-glycome in GDM. We were able to reliably quantify N-glycome composition of these glycoproteins in pregnant women burdened with GDM and compare it to the pregnant women with normal glycemic status. Our results showed a lack of significant alterations in N-glycome composition related exclusively to GDM, however, numerous N-glycosylation features were vastly associated with markers of metabolic health in pregnancy.

Firstly, we profiled plasma protein N-glycome, which predominantly consisting of biantennary, triantennary and tetraantennary complex type N-glycans. All these N-glycans contain a heptasaccharide core which may carry additional *N*-acetylglucosamine(s) (GlcNAc), bisecting GlcNAc, antennary and/or core fucose, galactose(s) and sialic acid(s). Human plasma is a complex mixture of proteins, whose glycosylation profile is typically dominated by a smaller number of highly abundant glycoproteins. Besides, plasma protein N-glycan pattern predominantly reflects plasma cell- and hepatocyte-specific (dys)regulation of glycosylation, conferring important information on inflammatory and metabolic status of an individual. Our data show that numerous plasma protein N-glycans associate with markers of metabolic status, such as fasting glucose and insulin levels, markers of insulin resistance and lipids, even though we observed no hepatic dysfunction in our subjects. Namely, the most pronounced (negative) associations were identified between fasting insulin levels (and accordingly, between estimators of insulin resistance and β -cell function) and various N-glycan structures bearing bisecting GlcNAc. A significant decrease in bisection of biantennary glycans has been previously described in type 2 diabetes [22], but the exact functional implications of this glycosylation alteration in diabetes are unknown. In general, bisecting GlcNAc confers unique lectin recognition properties and in this way restricts the mobility of the carrying glycoprotein [23]. A decrease in levels of bisecting GlcNAc has been observed during enhanced stimulation of cells with insulin and activation of insulin receptor and IGF-I receptor signalling [24], revealing that bisection might have a role in insulin signalling pathways. Nonetheless, additional studies are warranted to unravel the exact underlying mechanisms and whether the observed changes in bisection could indicate an early-stage dysregulation of insulin action. Since every pregnancy is characterized by a certain degree of insulin resistance, we have also noted a negative association of insulin levels and insulin resistance markers with low-branched glycans and a positive one with high-branched glycans. The concomitant increase in plasma protein high-branched and decrease in low-branched glycans is typically seen in type 2 diabetes [22, 25, 26], and is usually attributed to the increased glucose flux and its utilization by the hexosamine biosynthetic pathway. Our results showed that such glycosylation changes are notable even in pregnant women with normal glucose tolerance, suggesting that plasma protein glycosylation reflects metabolic changes related to insulin resistance and β-cell (dys) function at their earliest, along the glycaemic continuum. When examining the associations between lipid markers in pregnancy and plasma protein glycosylation, we obtained the similar results for both total cholesterol and triglycerides, which is not unexpected, as their increased levels reflect the poorer metabolic health. Specifically, we observed a positive association between the marker levels and high mannose glycans, which are known to predominantly originate from apolipoprotein B-100 [27], a major component of LDL particles and regulator of LDL cholesterol homeostasis in the plasma [28]. Additionally, lipid markers positively associated with abundantly galactosylated and sialylated complex N-glycans, which is in line with a previous report [29].

Next, we examined protein-specific N-glycosylation profiles, to check if identified associations correlate with overall glycosylation changes on all plasma proteins or if they are protein-specific and have a potential implication on protein function. The most abundant antibody in the human blood plasma is IgG, which mainly carries biantennary complex type N-glycans in the heavy chain constant region of the fragment crystallizable (Fc) domain. Around 20% of IgG molecules also carry N-glycan on fragment antigen-binding (Fab) domain. Our data revealed that metabolic parameters related to insulin levels and insulin resistance significantly associate with two distinct N-glycan structures—FA2B and A2G2S2. Herein, the FA2B structure negatively associates with insulin levels, but the same structure has been previously found to be increased in type 2 diabetes [30, 31]. This might be attributed to the fact that type 2 diabetes pathogenesis, apart from insulin resistance, also involves defects of insulin-producing β-cells, responsible for decreased insulin production and secretion. Considering well-established link between hyperglycemia in pregnancy and type 2 diabetes, our results indicate that glycan structures may reflect very early changes in the impaired glucose homeostasis. Conversely, IgG N-glycan A2G2S2 has not been previously linked to diabetes nor insulin resistance. It is thought that placental-derived hormones are a major factor in reprogramming maternal physiology to achieve an insulin-resistant state during pregnancy [32]. In type 2 diabetes, insulin resistance is characterized by chronic low-grade inflammation and infiltration of various immunocompetent cells into visceral adipose tissue. Previous mouse model studies have revealed that, for instance, infiltrating B-lymphocytes exacerbate metabolic disease by producing pathogenic IgGs, that induce insulin resistance through an Fc receptor-mediated process [33]. It was later demonstrated that only a distinct IgG glycoform—hyposialylated IgG, is likely responsible for obesity-related genesis of insulin resistance, driven by enhanced activation of endothelial FcγRIIB receptor, which consequently impairs insulin delivery to the skeletal muscle [34]. Herein, we observed a positive association between IgG sialylation levels and HOMA2-%B index, suggesting that IgG might also play a role in the induction of β -cell dysfunction in pregnancy. This is further corroborated by a previous study which demonstrated that pregnancy alone was associated with a marked reduction (40%) in insulin-stimulated glucose transport, despite the absence of any detectable change in total GLUT4 abundance in skeletal muscle [35]. Metabolic parameters describing lipid status mostly associated with glycan traits describing IgG galactosylation and incidence of high mannose structures. It has been reported previously that total cholesterol did not significantly influence IgG glycosylation, whereas an increase in IgG galactosylation and sialylation has been observed at low levels of triglycerides [36]. Of note, the study [36] examined Fc IgG glycans only, while herein we investigated both Fc and Fab IgG glycans.

Subsequently, we examined N-glycosylation profile of circulating IgA, the second most abundant immunoglobulin in human plasma, which mainly carries biantennary complex type N-glycans and low amounts of triantennary N-glycans. We found that fasting glucose levels negatively associate with levels of IgA bisection, however, the exact functional role of bisecting GlcNAc on IgA is unknown. Combining of these findings with observations on associations between plasma protein bisection and insulin levels suggests that bisecting GlcNAc might confer functions relevant for maintenance of glucose homeostasis, through mechanisms yet to be elucidated. Insulin level and insulin resistance indices showed positive association with the same triantennary fully sialylated N-glycan (A3G3S3), while HOMA2-%B index additionally exhibited positive association with glycan A2G2S2, which was also positively associated with insulin levels and insulin resistance/β -cell function indices when originating from IgG. In general, IgA glycome showed the most extensive associations to the total cholesterol levels, where the incidence of high mannose glycans and digalactosylation seem to be the most prominently linked features. Implicated biological pathways that would explain the observed associations have yet to be resolved, since, in general, there are hardly any studies examining IgA N-glycosylation in dyslipidemia, diabetes or any other metabolic disorder.

Furthermore, this study is the first to compare plasma protein, IgG and circulating IgA N-glycomes between pregnancies burdened with GDM and pregnancies with normal glucose tolerance. None of the tested glycan structures or glycosylation features showed significant alterations related to GDM and none were able to differentiate pregnant women with normal glucose tolerance from those with GDM. This could be due to the fact that pregnancy itself encompasses extensive metabolic changes, including a 50%-reduction of peripheral insulin sensitivity and a 2-3-fold increase in insulin secretion [6], which may impact protein N-glycosylation at a comparable level in normoglycemic and GDM pregnancies. Our recruitment strategy may have had an impact as well, since we sampled participants during oral glucose tolerance testing, i.e., at the time of diagnosis establishment, when the impact of GDM on protein glycosylation still might not have been so pronounced as it would be in the later stages of pregnancy. Moreover, pregnancy introduces not just metabolic, but numerous and extensive immune, hormonal and hematologic changes that could also affect protein glycosylation, potentially masking the impact of GDM alone. Various changes in plasma protein, IgG and circulating IgA N-glycosylation during pregnancy have been observed previously. For instance, plasma glycoproteins showed increased levels of largely sialylated bi-, tri-, and tetra-antennary glycans during pregnancy, thought to be involved in the regulation of pro- and anti-inflammatory immune responses, essential for maternal-fetal tolerance [37, 38]. Furthermore, pregnancy leads to an increase in IgG galactosylation and sialylation, two IgG glycosylation features known to play an anti-inflammatory role [39–41]. As for the plasma-derived IgA, one study reported no significant pregnancy-associated glycosylation changes [38], while another reported a glycosylation site-specific increase in IgA bisection and sialylation during pregnancy [42], but whether the changes are biologically important needs to be further investigated. Only one study reported the glycosylation alterations of sIgA (isolated from human milk) related to GDM, where a decrease in high mannose, fucosylated and sialylated sIgA N-glycans was observed [16], indicating that the impact of GDM on IgA glycosylation may be detectable only in the postpartum period or on sIgA. Finally, diagnostic criteria for GDM used in this study may also have had an impact on the results, considering a stringent fasting plasma glucose cut-off set at 5.1 mmol/L which is based on the perinatal outcomes (2) and far below normoglycaemic cut-off for non-pregnant individuals set at 6,1 mmol/L [43]. However, recently published follow-up study revealed that GDM, diagnosed according to IADPSG/WHO criteria was significantly associated with a higher maternal risk for type 2 diabetes and prediabetes long-term after pregnancy [44]. On the other side, childhood insulin resistance with a limited β -cell compensation and impaired glucose tolerance was found to be independently associated with an exposure to untreated GDM *in utero* [45]. These evidence not only confirm the validity of the applied diagnostic criteria in detecting disturbed glucose homeostasis in pregnancy with a potential transgenerational impact, but also provide a valid methodological approach in studying various components of the complex pathophysiology of hyperglycaemia, with N-glycome aberrations as a plausible research goal.

Lastly, we want to acknowledge several limitations of our study. As a pilot study, we were limited by the size of our study population, so certain effects may not be visible. Moreover, our analytical approach relatively quantifies released glycan species, thus in the case of total plasma protein glycome, we’re missing the information about the source proteins of distinct glycan structures. Thus, the observed plasma protein glycosylation alterations could be attributed to both variation in protein concentration or to variation in glycan occupancy on certain proteins. Also, two glycan peaks contain coeluting structures with similar abundancies, hence the major glycan structure could vary between individuals. Lastly, the ratios of distinct glycan structures may be affected by different proportions of immunoglobulin subclasses, although we found no literature data on changes in immunoglobulin subclass ratios related to GDM or within the same trimester of pregnancy.

## Conclusion

To summarize, our results demonstrate that many metabolic markers that assess glucose tolerance in pregnancy show extensive associations to numerous N-glycosylation features. However, plasma protein, IgG and IgA N-glycans were not able to differentiate pregnant women with normal glucose tolerance from those with GDM, probably due to numerous physiological changes that accompany pregnancy, which potentially confound the impact of GDM on protein glycosylation. Nonetheless, since the factors that underlie chronic insulin resistance in former GDM patients remain to be identified [32], our findings may direct future research in finding additional biomarkers that predict pregnancy complications, as the multiple features of protein glycosylation have the advantage of providing more information than the level of a single analyte/marker.

## Supporting information

S1 Table Detailed description of glycan structures corresponding to every individual plasma protein, IgG and IgA glycan peak (GP). (XLSX)

S2 Table Derived glycan traits calculated from initial glycan peaks. (XLSX)

S3 Table Associations between plasma protein N-glycan traits and metabolic markers in pregnancy. (XLSX)

S4 Table Associations between IgG N-glycan traits and metabolic markers in pregnancy. (XLSX)

S5 Table Associations between IgA N-glycan traits and metabolic markers in pregnancy. (XLSX)

S6 Table Comparison of plasma protein, IgG and IgA glycan traits between pregnant women with normal glucose tolerance and pregnant women with gestational diabetes. (XLSX)

S7 Table Normalized glycan peak areas, and relevant clinical and biochemical data. (XLSX)

S1 File (DOCX)
