# Supplementary material for: Associations between plasma protein, IgG and IgA N-glycosylation and metabolic health markers in pregnancy and gestational diabetes
Source: PLoS One. 2023 Apr 20;18(4):e0284838. doi: 10.1371/journal.pone.0284838 (PMC10118201; doi:10.1371/journal.pone.0284838)
Supplement: S1 File — (DOCX) [file pone.0284838.s008.docx]

**Supporting Material**

**Associations between plasma protein, IgG and IgA N-glycosylation and metabolic health markers in pregnancy and gestational diabetes**

Tamara Štambuk^1,2^, Domagoj Kifer^1^, Lea Smirčić-Duvnjak^3^, Marijana Vučić Lovrenčić^3^, Olga Gornik^1^

^1^ Faculty of Pharmacy and Biochemistry, University of Zagreb, Zagreb, Croatia

^2^ Genos Glycoscience Research Laboratory, Zagreb, Croatia

^3^ Vuk Vrhovac University Clinic for Diabetes, Endocrinology and Metabolic Diseases, Merkur University Hospital, University of Zagreb School of Medicine, Zagreb, Croatia


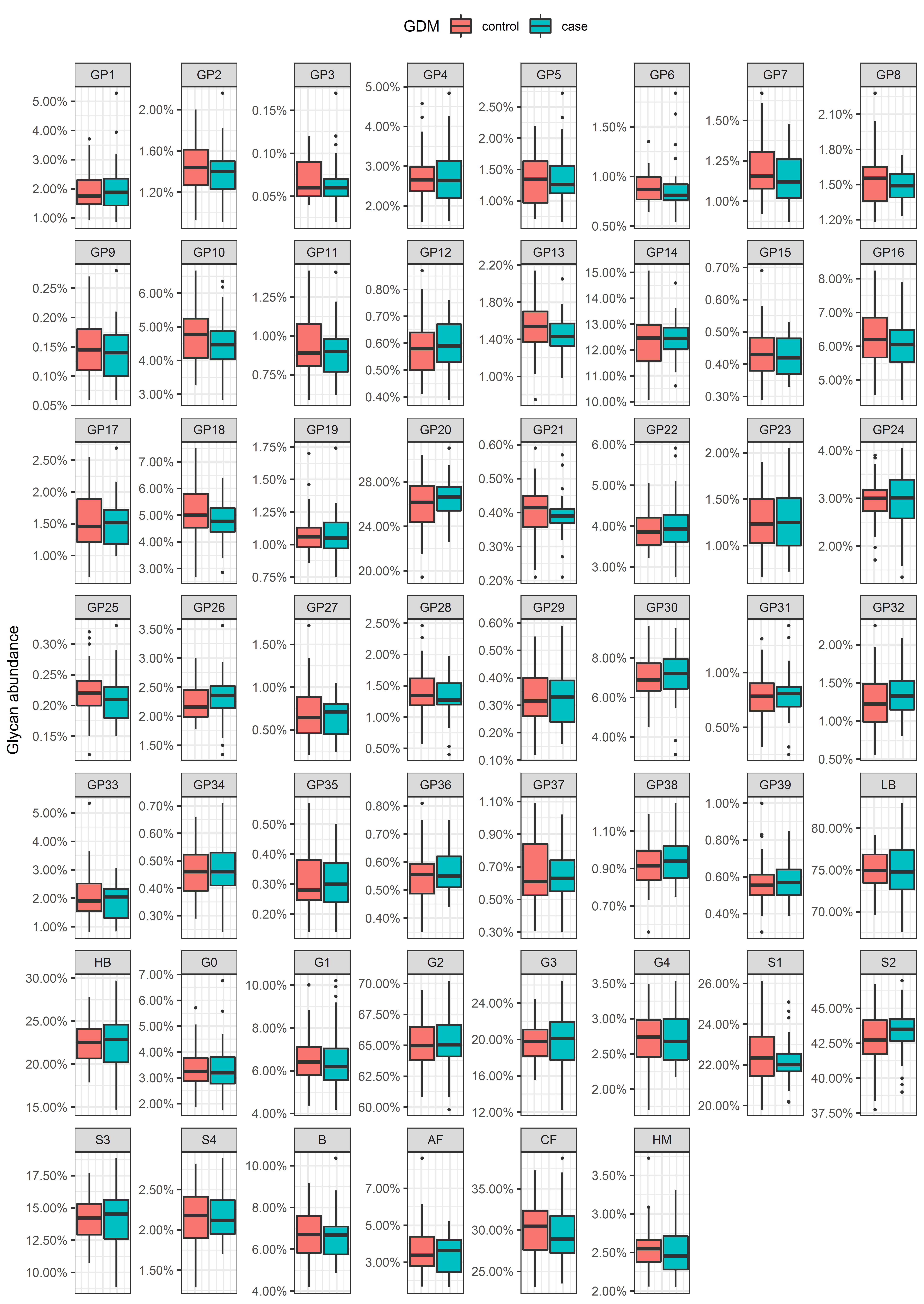


**S1 Figure. Differences in abundance of plasma protein N-glycan traits between pregnant women with normal glucose tolerance and pregnant women with gestational diabetes.** Differences in glycan abundances are shown as box plots. Each box represents the 25th to 75th percentile. The upper whisker extends from 75th percentile to the values within 1.5 x IQR (where IQR is the inter-quartile range, or distance between the first and third quartiles). The lower whisker extends from 25th percentile to the values within 1.5 x IQR. Dots indicate outliers.


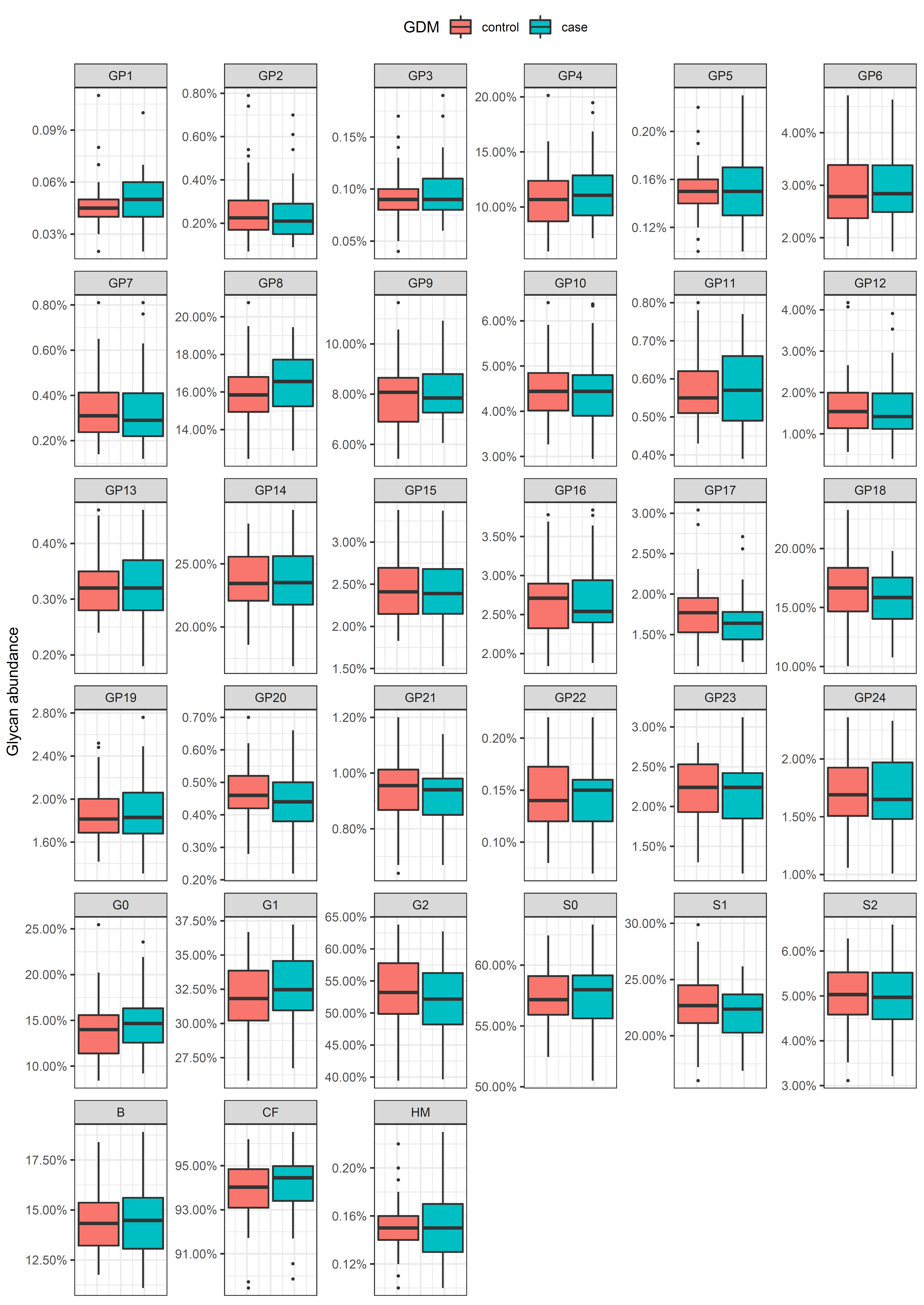


**S2 Figure. Differences in abundance of IgG N-glycan traits between pregnant women with normal glucose tolerance and pregnant women with gestational diabetes.** Differences in glycan abundances are shown as box plots. Each box represents the 25th to 75th percentile. The upper whisker extends from 75th percentile to the values within 1.5 x IQR (where IQR is the inter-quartile range, or distance between the first and third quartiles). The lower whisker extends from 25th percentile to the values within 1.5 x IQR. Dots indicate outliers.


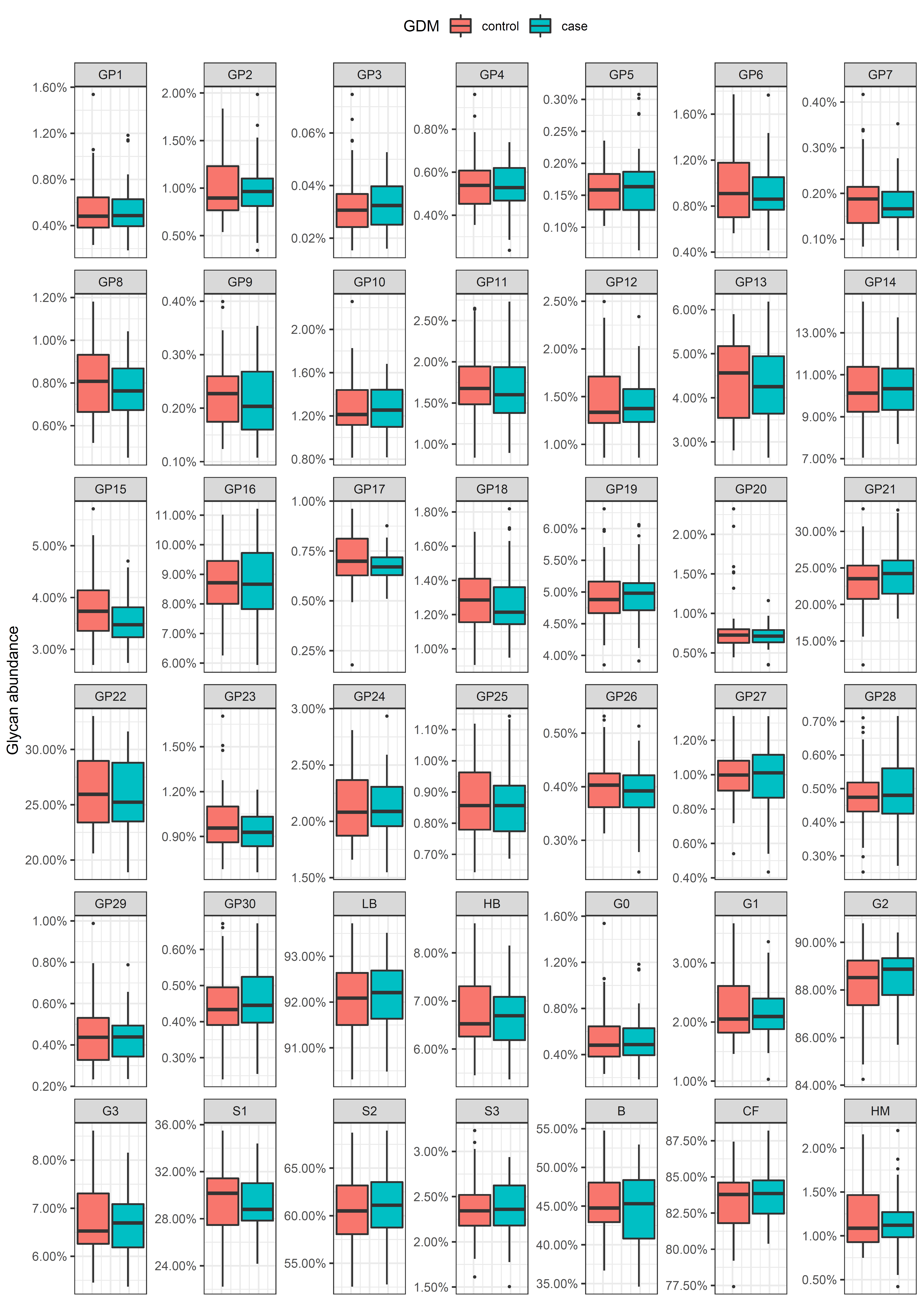


**S3 Figure. Differences in abundance of IgA N-glycan traits between pregnant women with normal glucose tolerance and pregnant women with gestational diabetes**. Differences in glycan abundances are shown as box plots. Each box represents the 25th to 75th percentile. The upper whisker extends from 75th percentile to the values within 1.5 x IQR (where IQR is the inter-quartile range, or distance between the first and third quartiles). The lower whisker extends from 25th percentile to the values within 1.5 x IQR. Dots indicate outliers.
